# Supplementary material for: Tuning orbital-selective phase transitions in a two-dimensional Hund’s correlated system
Source: Nat Commun. 2023 Jun 16;14:3572. doi: 10.1038/s41467-023-39188-9 (PMC10275918; doi:10.1038/s41467-023-39188-9)
Supplement: Supplementary file 1 — Supplementary information [file 41467_2023_39188_MOESM1_ESM.pdf]

Supplementary Information

Tuning orbital-selective phase transitions in a two-dimensional Hund's correlated system

Eun Kyo Ko[1,2†], Sungsoo Hahn[1,2†], Changhee Sohn[3], Sangmin Lee[4], Seung-Sup B. Lee[2], Byungmin Sohn[1,2], Jeong Rae Kim[1,2], Jaeseok Son[1,2], Jeongkeun Song[1,2], Youngdo Kim[1,2], Donghan Kim[1,2], Miyoung Kim[4], Choong H. Kim [1,2*], Changyoung Kim[1,2*], Tae Won Noh[1,2*]

[1]*Center for Correlated Electron Systems, Institute for Basic Science (IBS), Seoul 08826, Republic of Korea*

[2]*Department of Physics and Astronomy, Seoul National University, Seoul 08826, Republic of Korea*

[3]*Department of Physics, Ulsan National Institute of Science and Technology, Ulsan, Republic of Korea*

[4]*Department of Materials Science and Engineering and Research Institute of Advanced Materials, Seoul National University, Seoul 08826, Republic of Korea*

[†]These authors contributed equally.

[*]e-mail: chkim82@snu.ac.kr, changyoung@snu.ac.kr, twnoh@snu.ac.kr

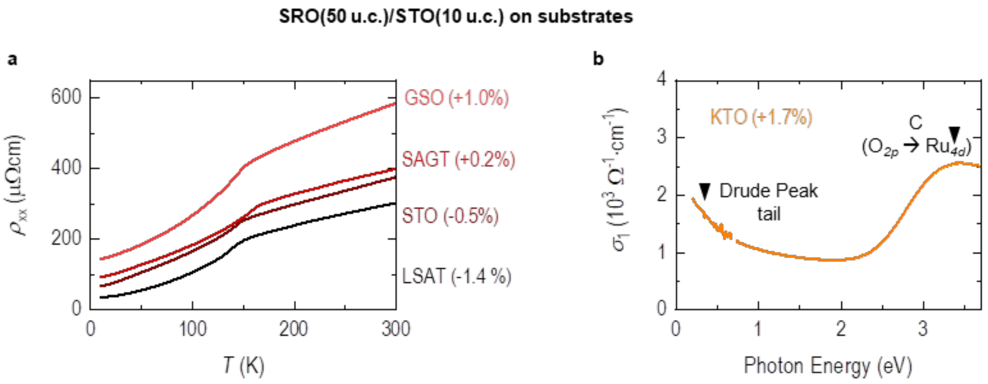

Supplementary Fig.1 | Metallic states of thick $SrRuO_3$ (SRO) films. a, Temperature-dependent longitudinal resistivity (ρ_{xx}) of a 50unit cell (u.c.) SRO/10 u.c. $SrTiO_3$(STO) film on various substrates. Compressive (tensile) strain is indicated by the minus (plus) sign. The strain is calculated in reference to the lattice constant of bulk SRO. All samples behave as metals. **b,** Optical spectrum of SRO (50 u.c.) deposited on a $KTaO_3$ (KTO) substrate (+1.7%). Measurement of the transport properties of SRO (50 u.c.) on the KTO substrate was challenging because the large tensile strain created cracks in thick films[1], rendering transport path discontinuities. Instead, we measured the optical spectra (in the infrared and ultraviolet regions) via ellipsometry. A Drude peak for the thick film under +1.7% strain is apparent, indicating a metallic state.

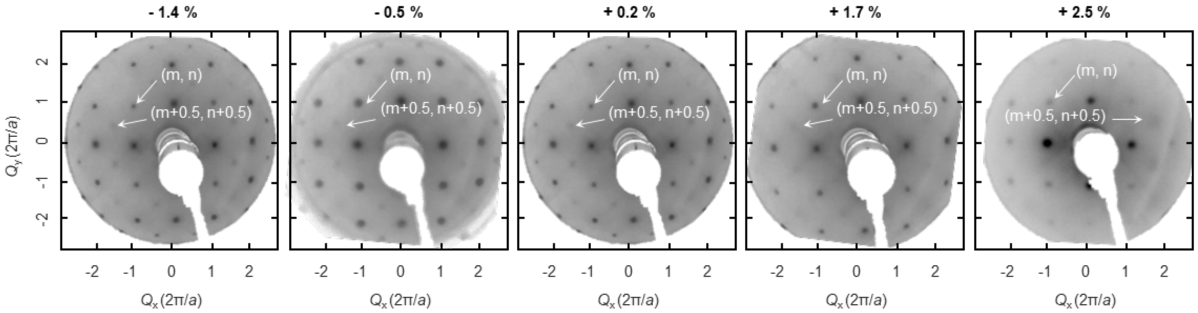

Supplementary Fig.2 | Structural characterization of a SRO monolayer via low-energy electron diffraction (LEED). LEED peaks are denoted by (m, n) where m and n are integers. If there is $\sqrt{2} \times \sqrt{2}$ reconstruction due to the rotation of RuO_6 (along the out-of-plane), (m+0.5, n+0.5) peaks should appear. On the other hand, 2×2 reconstruction due to tilting of RuO_6 (along the in-plane) should result in (m+0.5, n) or (m, n+0.5) peaks. LEED images of our SRO monolayers on various substrates were captured at 200 eV and 6 K. All samples exhibit (m+0.5, n+0.5) peaks without (m+0.5, n) and (m, n+0.5) peaks. Therefore, we can conclude that the structural symmetries of the SRO monolayers are identical, independent of the applied epitaxial strain and substrates. The preserved symmetry is attributable to the inserted STO layers[2].

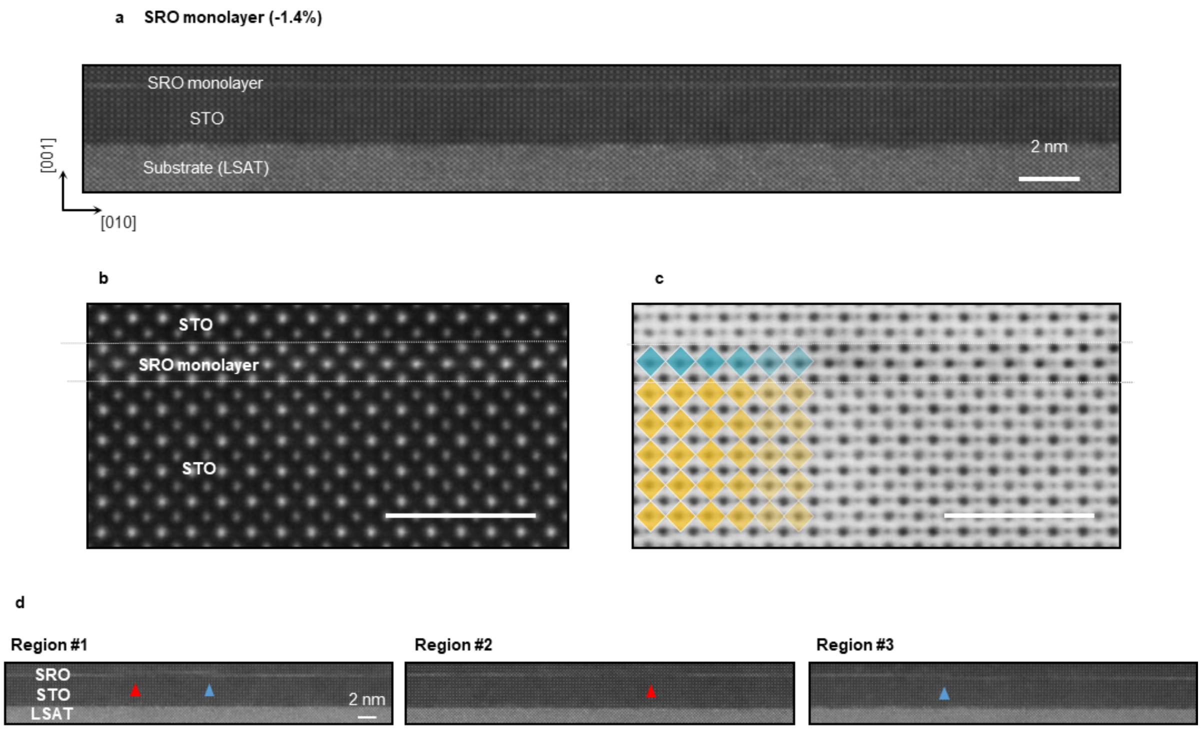

Supplementary Fig.3 | Scanning transmission electron microscopy (STEM) images of SRO on LSAT(001). a, Structural characterization of an SRO monolayer on a STO (10 u.c.)-LSAT substrate via high-angle annular dark-field (HAADF) mode. A 10 u.c. STO cap protects the SRO from damage during measurements. **b, c** HAADF (**b**) and annular bright-field images (**c**). A single RuO_2 layer with abrupt interfaces and without RuO_6 octahedron tilting is revealed. **d,** HAADF-STEM images of different regions in SRO on LSAT(001). Most regions have atomically sharp interfaces with the SRO monolayer, as shown in **Fig. 2** (main text). However, a few regions exhibit thickness inhomogeneities. The red arrows indicate regions with discontinuous RuO_2 layers (0 u.c. SRO). The blue arrows indicate regions with RuO_2 double layers (2 u.c. SRO).

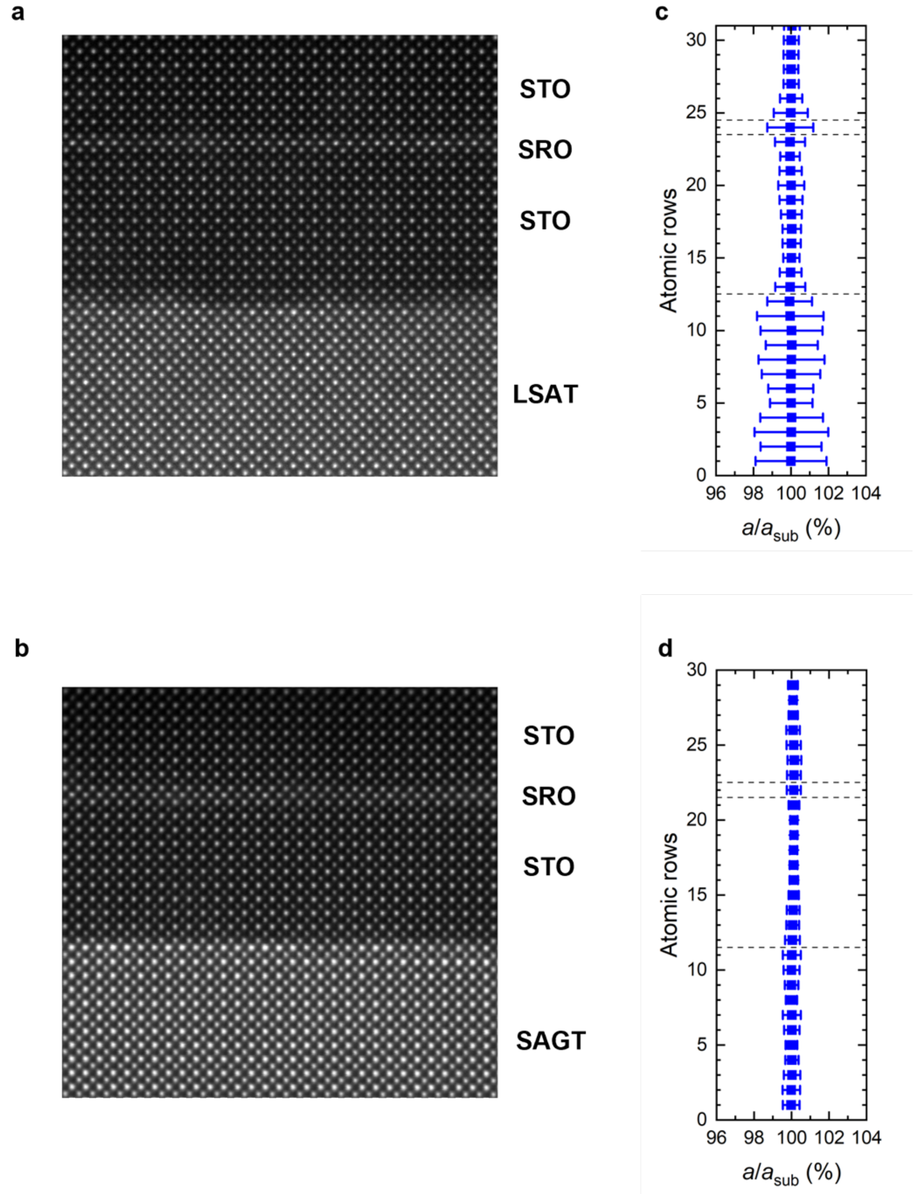

Supplementary Fig.4 | Lattice constant analysis from STEM results for STO(10 u.c.)/SRO(1 u.c.)/STO(10 u.c.) on LSAT(001) and SAGT(001). a,b, STEM-HAADF images of STO(10 u.c.)/SRO(1 u.c.)/STO(10 u.c.) on LSAT(001) (**a**) and SAGT(001) (**b**). **c,d,** In-plane lattice constant as a function of the atomic row number. Both SRO and STO layers are fully strained to the substrate.

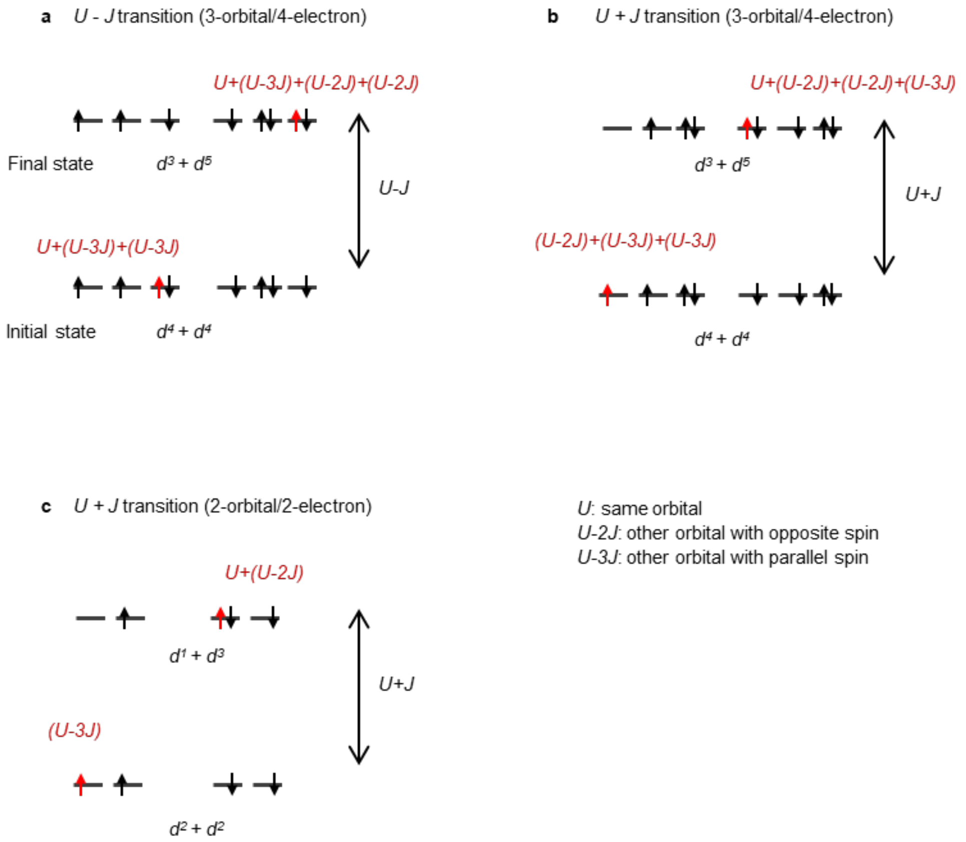

Supplementary Fig.5 | Interatomic *d-d* transitions in ruthenates. a, b, Interatomic *d-d* transition with an energy cost of $U - J$ (**a**) and $U + J$ (**b**) in three-orbital/four-electron (hereafter, 3-orbital/4-electron) systems. **c,** Interatomic *d-d* transition with an energy cost of $U + J$ in two-orbital/two-electron (hereinafter, 2-orbital/2-electron) systems.

The interaction between electrons in the same orbital is represented by U. When the rotational symmetry of the Hamiltonian for t_{2g} orbitals is considered, interactions between electrons in different orbitals (i.e., with opposite spin) can be described by $U - 2J$, where J is the Hund's rule coupling. Interactions between electrons in different orbitals with the same spin can be described by $U - 3J$. The energy difference between the initial ($d^4 + d^4$) and final ($d^3 + d^5$) states is the energy cost of the transition.

Let us consider the $d^4 + d^4 \rightarrow d^3 + d^5$ transition for a material with a (3-orbital/4-electron) system. Here, we consider only cases with paramagnetic or antiferromagnetic

ordering. Due to the Pauli exclusion principle, electrons with the same spin cannot occupy the same orbital state. Thus, there should be two kinds of interatomic transitions. Given J, they have different energies: $U - J$ and $U + J$.

When Δ_t is large, d_{xy} is fully occupied, which effectively represents a (2-orbital/2-electron) system. In such a case, only the $U + J$ transition occurs because all involved orbitals are half filled (under the influence of J).

The results shown in **Fig. 3** (main text) confirm the role played by J in electron distributions. Assuming that there is no J for an effective 2-orbital/2-electron system, the electron configuration may be, for example, (↑↓, 0), (0, ↑↓), (↑, ↑). In such cases, fully-filled orbitals whose nearest-neighbor ions have half-filled (or empty) orbitals will induce peak A ($U - J$). However, as shown in **Fig. 3**, the $U - J$ transition disappears for our SRO under strain, indicating the existence of strong J effects. In other words, following the Hund's rule, the two electrons are distributed equally in the two orbitals, preferring the high-spin configuration.

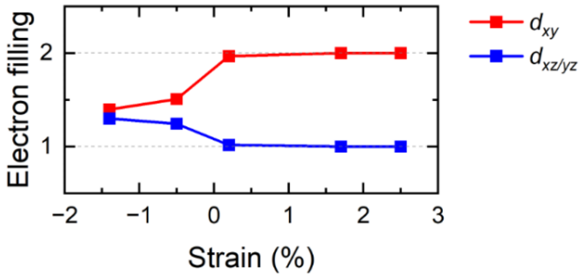

Supplementary Fig.6 | Strain-dependent electron filling calculated via DMFT with *U* = 2.7 eV and *J* = 0.45 eV.

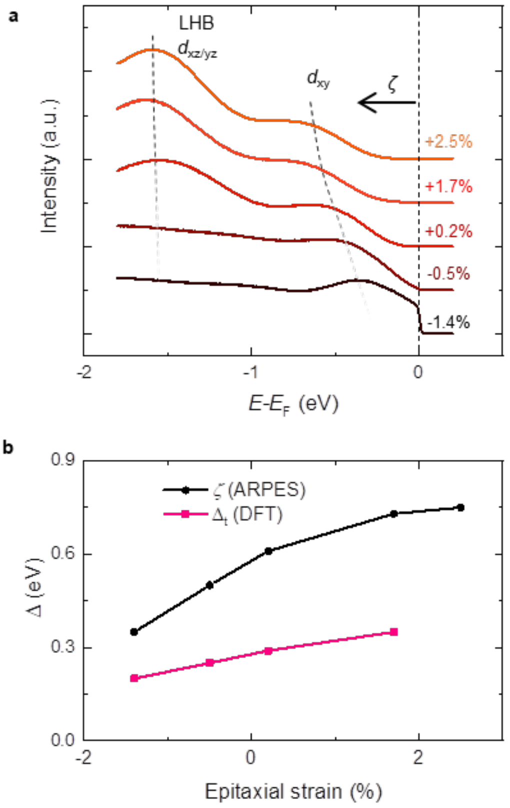

Supplementary Fig.7 | MIT with Δ_t modulation. a, EDCs from the Γ-M cut for SRO monolayers under −1.4, −0.5, +0.2, +1.7, and +2.5% strains. The band near M/2 has a d_{xy} character, whereas that near Γ has a $d_{xz/yz}$ character. **b,** Strain-dependent energy position of the d_{xy} band (ζ) and Δ_t calculated via density functional theory (DFT).

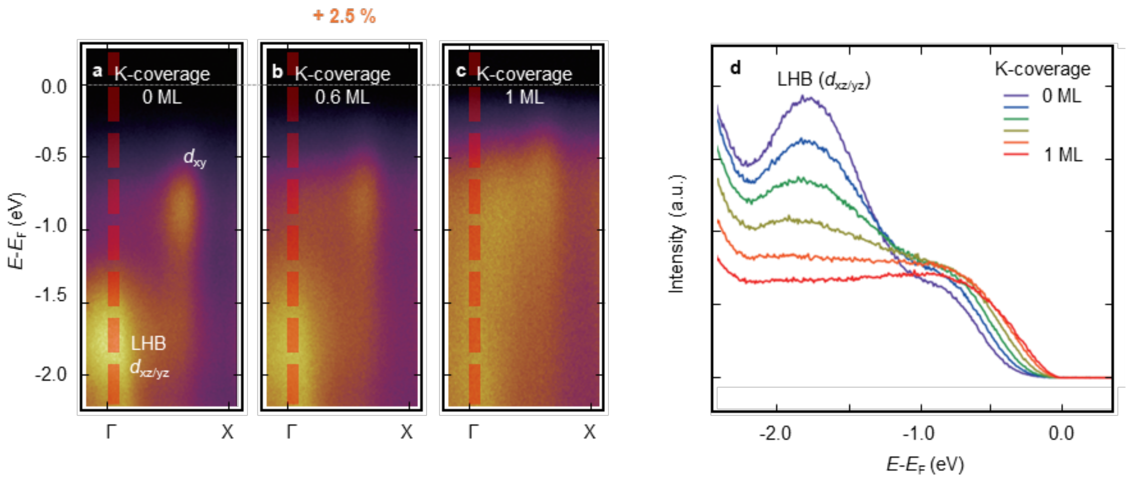

Supplementary Fig.8 | Doping-dependent studies of an effective (2-orbital/2-electron) system. **a-c,** *E-k* data from an SRO monolayer with +2.5% strain along the Γ-X line. The K-coverage ranges from 0.0 monolayer (ML) to 1.0 ML. **d,** EDCs near the Γ point (thick red dashed lines in **a–c**) over the range of $0.0\ \text{Å}^{-1} \le k_x \le 0.1\ \text{Å}^{-1}$ and $k_y = 0.0\ \text{Å}^{-1}$.

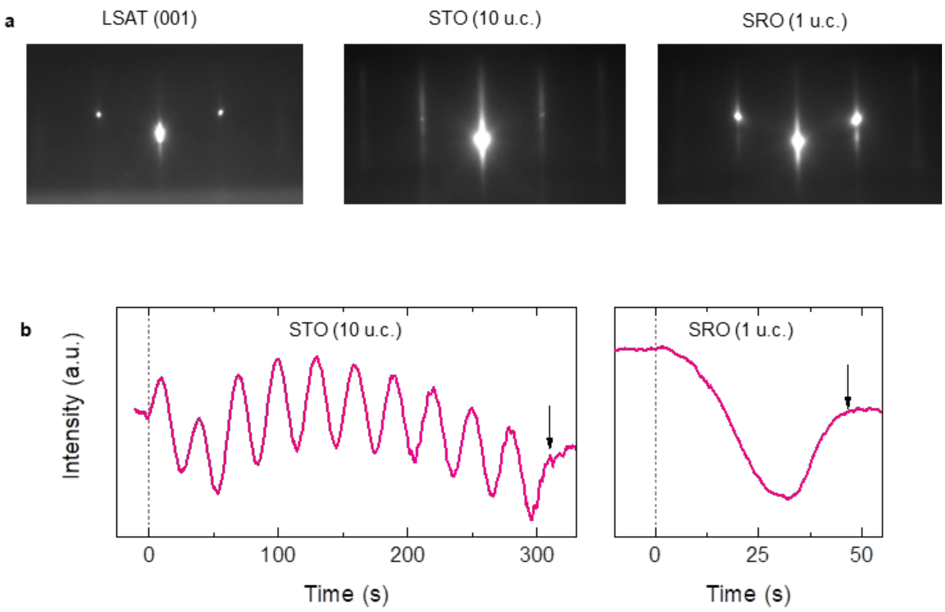

Supplementary Fig.9 | Reflection high-energy electron diffraction (RHEED) images taken during deposition. a, RHEED patterns of the LSAT(001) substrate, STO (10 u.c.) film, and a monolayer SRO film along the $[100]_{\text{pseudo-cubic}}$ direction**. b,** RHEED intensities. On the left, a 10 u.c. STO layer is deposited on an LSAT substrate in layer-by-layer growth mode. On the right, 1 u.c. of $SrRuO_3$ (SRO) is deposited on a STO (10 u.c.)-LSAT substrate.

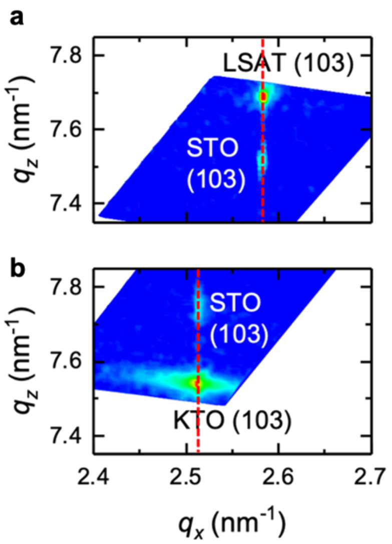

Supplementary Fig.10 | XRD scans for STO (10 u.c.)/SRO (1 u.c.)/STO (10 u.c.)/SRO (4 u.c.)/STO (10 u.c.) on LSAT(001) and KTO(001) substrates. Reciprocal space mapping scans of the heterostructures on LSAT(001) **(a)** and KTO(001) **(b)**, with -1.4 % and +1.7% strains, respectively. The red dashed lines indicate the q_x value of the substrate (103) peak. The in-plane lattice constants of STO layers well match those of the substrates, indicating films are fully strained to the substrates.

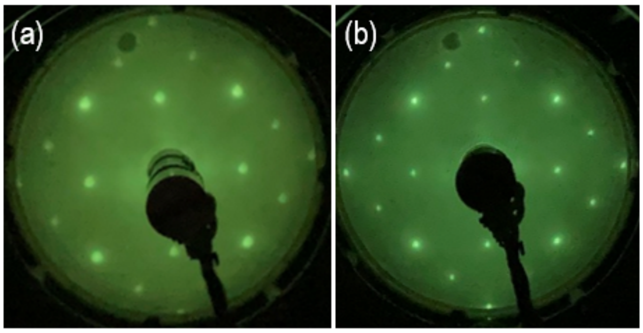

Supplementary Fig.11 | Surface structure characterization of monolayer SRO using LEED experiment. LEED pattern of monolayer SRO measured (a) before, and (b) after post-annealing at 570 °C for 20 min. The LEED images were taken with an electron energy of 110 eV at 300 K.

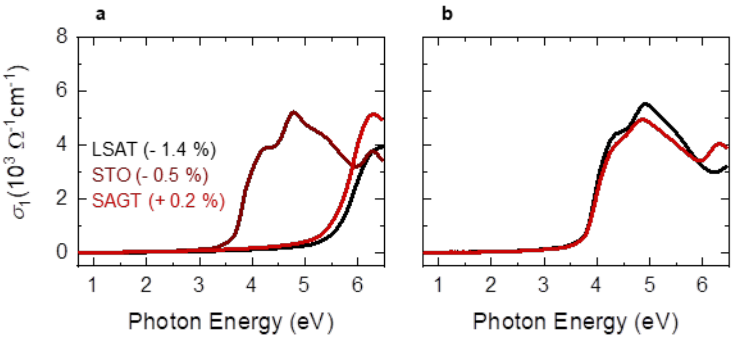

Supplementary Fig.12 | Optical spectra of substrates and STO (10 u.c.) layers. a, Optical spectroscopy results for LSAT, STO, and SAGT substrates. **b,** Optical spectroscopy results for STO (10 u.c.) layers deposited on LSAT and SAGT substrates. Band-insulating gaps of about 3.7 eV are evident, consistent with bulk STO.

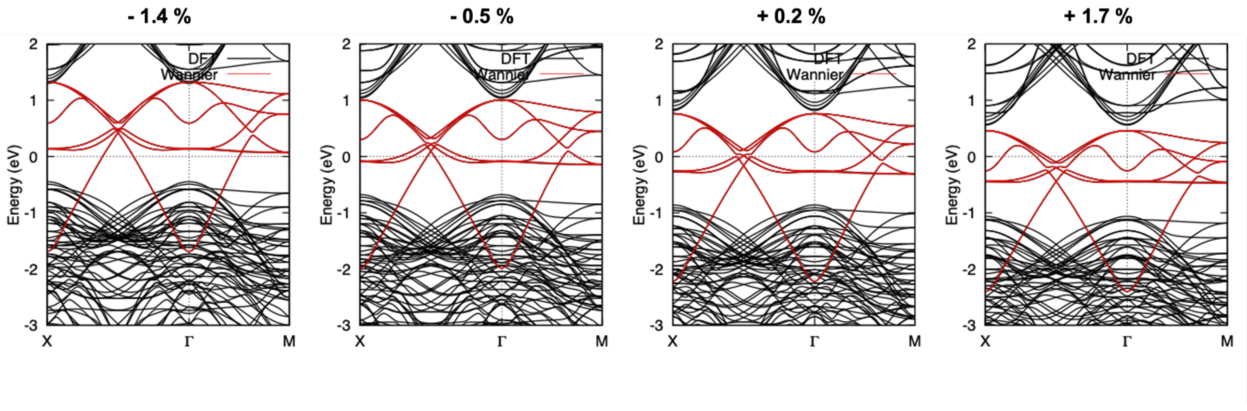

Supplementary Fig.13 | Electronic structure obtained from Wannierization of the t_{2g} orbitals for various strain values.

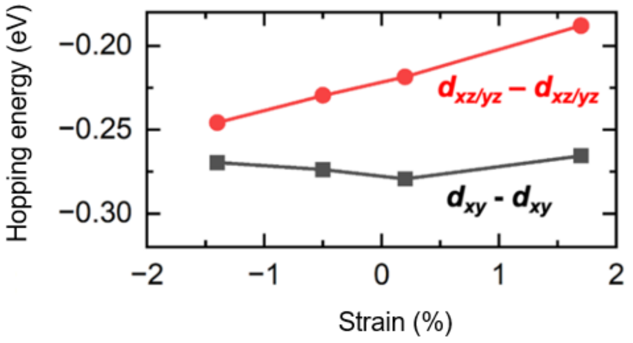

Supplementary Fig.14 | Orbital dependent hopping energy for various strain values.

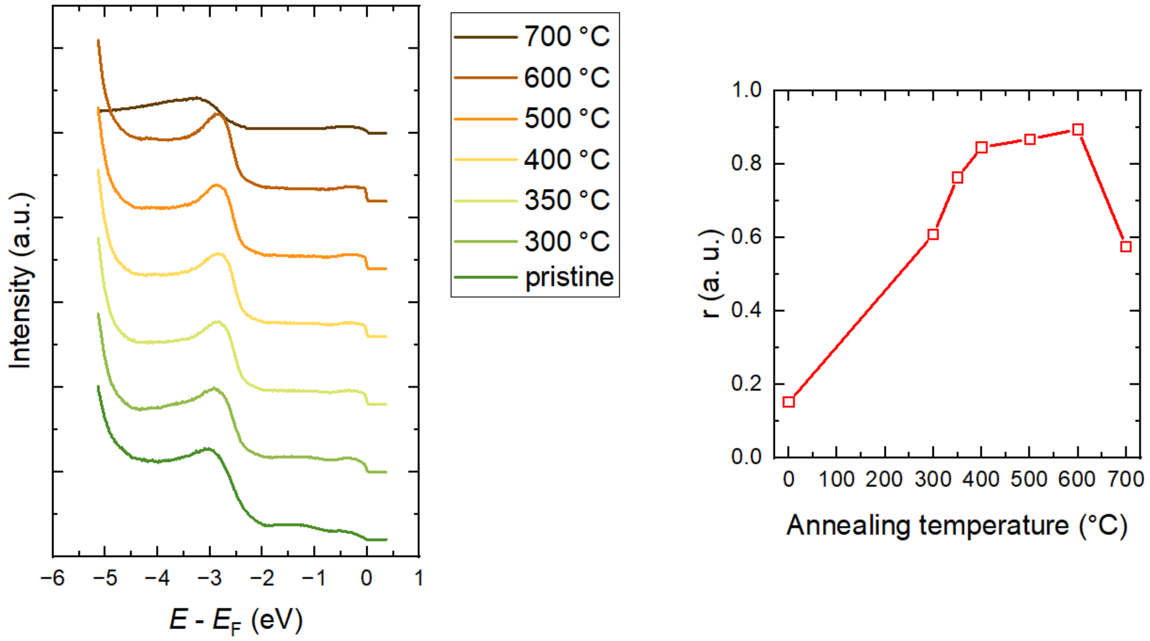

Supplementary Fig.15 | Annealing-temperature dependent photoemission spectra of $SrRuO_3$ (1 u.c.)/$SrTiO_3$ (10 u.c.)/$SrRuO_3$ (4 u.c.)/$SrTiO_3$ (10 u.c.) on LSAT(001). The samples were annealed at 300, 350, 400, 500, 600, and 700 °C in an ultrahigh vacuum (< 1.0 × 10^{-10} Torr) before photoemission measurements. The quasiparticle peak intensity ratio defined as $r = I(E - E_F = -0.05\ \mathrm{eV})/I(E - E_F = -0.4\ \mathrm{eV})$ is shown on the right.

Supplementary Note 1: possible contributions from the surface states to the spectroscopic results

The SRO monolayers were grown on STO (10 u.c.). Previous results have shown that STO surfaces with oxygen vacancies have metallic surface states[5,6]. On the other hand, Sohn *et al.* demonstrated that the surface of STO (10 u.c.) prepared under the opimum condition was insulating without surface states[7], indicating minimal oxygen vacancies in the top STO layer.

Next, let us discuss the influence of the surface condition of the SRO monolayer to spectroscopic results in terms of thickness inhomogeneity and surface termination. As shown in **Supplementary Fig. 3**, the SRO monolayer is mostly 1 u.c. thick. However, STEM results show thickness inhomogeneities in some regions (i.e., 0 or 2 u.c. of SRO). While such inhomogeneities can significantly affect certain experimental measurements such as transport properties, area-averaging techniques such as ARPES and optical spectroscopic experiments are less sensitive to such inhomogeneities. As our films are mostly 1 u.c. thick, the average spectroscopic response should represent that of an SRO monolayer.

The surface termination can induce changes in the electronic structure. For example, the oxygen octahedral symmetry may be broken at the surface if the film is B-site terminated[8]. However, SRO films grown by PLD are always Sr-terminated (A-site terminated) regardless of the substrate termination[9], due to the highly volatile Ru_xO_y at high temperature. Therefore, we can rule out the termination variation as the cause of the electronic structure change in SRO films.

Supplementary Note 2: azimuthal angle dependent APRES intensity

Note that the photoemission intensity is determined by the matrix element, i.e., $\langle f|\boldsymbol{A} \cdot \hat{\boldsymbol{p}}|i\rangle$ where $|i\rangle$ and $|f\rangle$ are the initial and final states of the photoelectron, respectively. $\boldsymbol{A}$ and $\hat{\boldsymbol{p}}$ are the vector potential and electron momentum operator, respectively. Let us consider the matrix element for the d_{xy} orbital. The symmetry of $|f\rangle$ is always even with respect to the plane of incidence. On the other hand, the symmetry of $|i\rangle$ in d_{xy} orbital is odd (even) for φ = 0° (45°). With *s* polarized light, the matrix element for the d_{xy} at φ = 45° should vanish. In other words, the ARPES intensity of d_{xy} with φ = 45° should be smaller than that with φ = 0°.

It is seen in **Fig. 5c** that the intensity along Γ-M for φ = 45° is significantly lower than that for φ = 0°. Therefore, the orbital character of the band at ≈–0.5 eV is of dominant d_{xy}. On the other hand, the data in **Fig. 5d** show that the intensity along Γ-M for φ = 45° is comparable to that for φ = 0°. This suggests dominant $d_{xz/yz}$ contribution to the band at ≈–1.6 eV.

Supplementary Note 3: the energy positions of LHB and UHB

The gap between lower Hubbard band (LHB) and upper Hubbard band (UHB) in our tensile-strained SRO monolayer can be different from *U* when we include the effect of *J*. In the 2-orbital/2-electron half-filled case, the Mott gap increases to $U + J$. The energy position of the LHB measured by ARPES is about 1.6 eV below E_F. In the case of a single-band system, this band will split symmetrically by $(U+J)/2$ around E_F. However, in multi-orbital systems, the presence of other bands makes the energy levels of LHB and UHB asymmetric around E_F. In the case of monolayer SRO, E_F is located between the d_{xy} band (not LHB) and UHB which correspond to the valence band top and conduction band bottom, respectively. In an analogous system of Ca_2RuO_4 which has the same orbital hierarchy and Mott insulating behavior, DMFT

results show that UHB is closer to E_F than LHB[10, 11].

References

1. Wang, H. *et al.* Direct Observation of Huge Flexoelectric Polarization around Crack Tips. *Nano Lett.* **20**, 88–94 (2020).

2. Kim, J. R. *et al.* Heteroepitaxial control of Fermi liquid, Hund metal, and Mott insulator phases in the single-atomic-layer limit. arXiv:2203.04244 (2022).

3. Moser, S. An experimentalist's guide to the matrix element in angle resolved photoemission. *J. Electron Spectros. Relat. Phenomena* **214**, 29–52 (2017).

4. Hu, B. *et al.* Surface and bulk structural properties of single-crystalline Sr 3Ru2O7. *Phys. Rev. B - Condens. Matter Mater. Phys.* **81**, 184104 (2010).

5. Soltani, S. *et al.*, $d_{xz/yz}$ orbital subband structures and chiral orbital angular momentum in the (001) surface states of $SrTiO_3$. *Physical Review B* **95**, 125103 (2017).

6. Walker, S. M. *et al.*, Control of a two-dimensional electron gas on $SrTiO_3$ (111) by atomic oxygen. *Physical Review Letter* **113**, 177601 (2014).

7. Sohn, B. *et al.*, Observation of metallic electronic structure in a single-atomic-layer oxide. *Nature Communications* **12**, 6171 (2021).

8. Lee, H. G. *et al.*, Atomic-scale metal-insulator transition in $SrRuO_3$ ultrathin films triggered by surface termination conversion. *Advanced Materials* **32** 1905815 (2020).

9. Koster, G. *et al.*, Structure, physical properties, and applications of $SrRuO_3$ thin films. *Reviews of Modern Physics* **84**, 253 (2012).

10. Ricco, S. *et al.*, In situ strain tuning of the metal-insulator-transition of Ca2RuO4 in

angle-resolved photoemission experiments. *Nature Communications* **9** 4535 (2018).

11. Sutter, D. *et al.*, Hallmarks of Hunds coupling in the Mott insulator Ca_2RuO_4. *Nature Communications* **8** 15176 (2017).
